# Type A aortic dissection during transoesophageal echocardiography: a case report

**DOI:** 10.1093/ehjcr/ytae413

**Published:** 2024-08-08

**Authors:** Chung-Yen Lee, Kuang-Chien Chiang, Wen-Jeng Lee, Chih-Yang Chan, Li-Tan Yang

**Affiliations:** School of Medicine, National Taiwan University, Taipei, Taiwan; School of Medicine, National Taiwan University, Taipei, Taiwan; Department of Medical Imaging, National Taiwan University Hospital, Taipei, Taiwan; Division of Cardiovascular Surgery, Department of Surgery, National Taiwan University Hospital, No. 7, Jhongshan S. Rd., Jhongjheng Dist., Taipei 10002, Taiwan; Division of Cardiology, Department of Internal Medicine, National Taiwan University Hospital, No. 7, Jhongshan S. Rd., Jhongjheng Dist., Taipei 10002, Taiwan; Department of Internal Medicine, College of Medicine, National Taiwan University, No. 7, Jhongshan S. Rd., Jhongjheng Dist., Taipei 10002, Taiwan; Telehealth Center, National Taiwan University Hospital, No. 7, Jhongshan S. Rd., Jhongjheng Dist., Taipei 10002, Taiwan

**Keywords:** Case report, Computed tomography, Type A aortic dissection, Transoesophageal echocardiography

## Abstract

**Background:**

The occurrence of type A aortic dissection (TAAD) during transoesophageal echocardiography (TEE) has only been reported once. We present another case of pre-procedural type B AD with retrograde TAAD or *de novo* TAAD during the TEE procedure.

**Case summary:**

An 81-year-old man with a pre-existing infrarenal abdominal aortic aneurysm and highly tortuous aorta was referred to our ward for acute decompensated heart failure (ADHF) with New York Heart Association functional class II. On hospital Day 2, the patient complained of intermittent dull pain over chest and back; ADHF or acute coronary syndrome was suspected. On Day 3, we transferred the patient to the intensive care unit due to ADHF with cardiogenic shock attributed to fluid overload, atrial fibrillation with rapid ventricular response, and severe mitral regurgitation with severely impaired left ventricular ejection fraction. Given the heightened surgical risk, TEE was performed to evaluate the eligibility of mitral transcatheter edge-to-edge repair. The first mid-oesophageal long-axis view showed no evidence of dissection. After 20 min, the same view showed the occurrence of TAAD. Urgent contrast CT confirmed a TAAD extending from the aortic root to the infrarenal abdominal aorta. Due to the prohibitive risk for surgical repair of TAAD, the patient received palliative care and unfortunately passed away on hospital Day 6.

**Discussion:**

Albeit rare, TAAD could progress or *de novo* occur during TEE, especially in high-risk patients. Therefore, high alertness during TEE procedures is imperative. Moreover, in patients with AD and poor renal function, the risk of using TEE as an alternative diagnostic modality should be carefully considered.

**Learning points**

- To acknowledge that aortic dissection (AD) could progress or occur *de novo* during a transoesophageal echocardiography (TEE) examination.
- In patients with deteriorated renal function and suspicion of an acute AD, physicians should carefully consider the risk of contrast-induced nephropathy from a CT scan and AD exacerbation during TEE when choosing diagnostic tools.

## Introduction

The occurrence of type A aortic dissection (TAAD) during transoesophageal echocardiography (TEE) has only been reported once.^1^ The patient received TEE for evaluating left atrial thrombi before catheter ablation, and the hypothesized mechanism was a hypertension surge.^1^ This report presents a tragic case with TAAD diagnosed during TEE for assessment of mitral transcatheter edge-to-edge repair (M-TEER) for severe functional mitral regurgitation (MR).

## Summary figure

**Figure:**
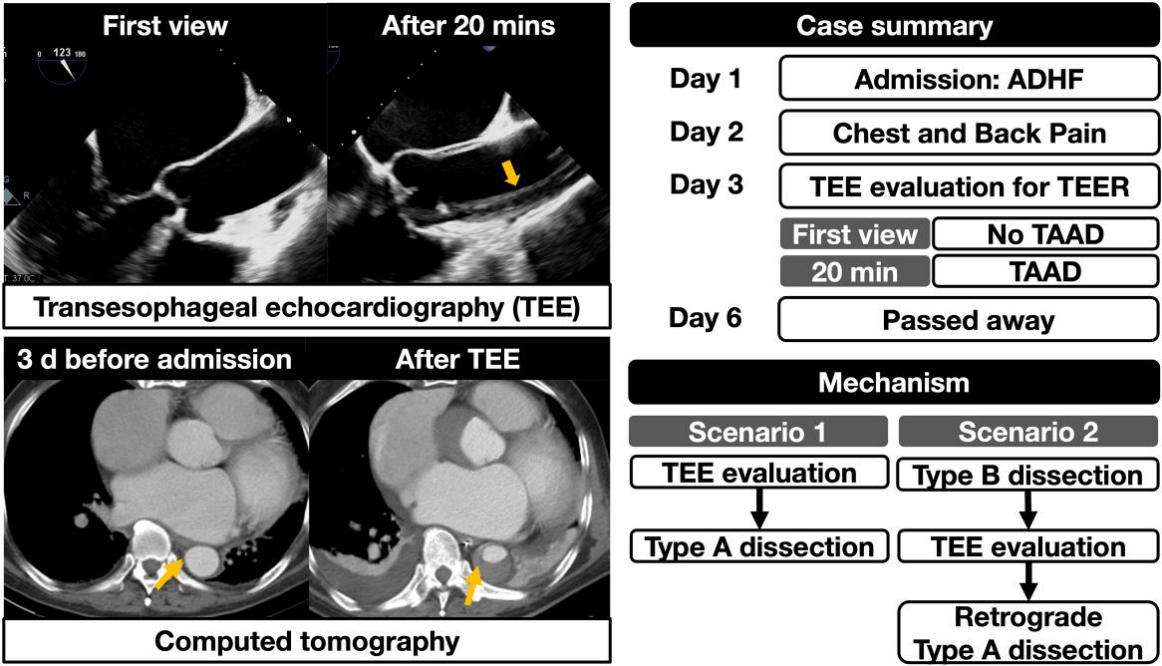


## Case presentation

An 81-year-old man visited a local hospital for progressive exertional dyspnoea, abdominal distension, and vomiting for 3 days. According to his family, he was a non-smoker and was diagnosed with heart failure (HF) with reduced ejection fraction 2 years ago. He was admitted for HF exacerbation later in the same year. He denied a past history of hypertension, diabetes, and hyperlipidaemia. Before this admission, he was New York Heart Association (NYHA) functional class II. Furosemide (40 mg b.i.d.), bisoprolol (1.25 mg b.i.d.), and amiodarone (100 mg b.i.d.) were prescribed 7 days before he visited the hospital.

Abdominal computed tomography (CT) was arranged and revealed an infrarenal abdominal aortic aneurysm (AAA) with a highly tortuous descending aorta (see Supplementary material online, *Video S1*). The patient was therefore referred to our emergency department with blood pressure (BP) 104/68 mmHg, heart rate (HR) 85 beats/min, respiratory rate (RR) 20 times/min, and peripheral oxygen saturation (SpO_2_) of 96% under 3 L/min O_2_ use. Cardiac auscultation revealed irregularly irregular heartbeats with an apical grade III/VI systolic murmur. An electrocardiogram revealed atrial fibrillation (AF). Chest X-ray showed cardiomegaly and mild pulmonary oedema. Bedside transthoracic echocardiography revealed a severely dilated left ventricle, severely reduced left ventricular (LV) ejection fraction (20.8%), and moderate to severe MR. Lab work revealed elevated creatinine 2.6 mg/dL (reference: 0.6–1.3 mg/dL) and poor renal function (estimated glomerular filtration rate: 25.3 mL/min/1.73 m^2^), N-terminal prohormone of brain natriuretic peptide 28 007 pg/mL (reference: < 125 pg/mL), and troponin-T 86.6 ng/L (reference: < 14 ng/L). Under the impression of acute decompensated HF (ADHF) with NYHA functional class II and infrarenal AAA, the patient was admitted to our surgical ward (hospital Day 1). Initial treatments included amiodarone (100 mg b.i.d.) for rhythm control and oral furosemide (40 mg b.i.d.) for fluid overload.

On Day 2, the patient reported intermittent dull pain over chest and back; ADHF or acute coronary syndrome was suspected. His vital signs showed BP 107/77 mmHg, HR 85 b.p.m., RR 18 times/min, and SpO_2_ 96% under 3 L/min O_2_ use. Electrocardiogram revealed AF with rapid ventricular response (AFRVR) and T wave inversion in leads V5–V6. Serial cardiac enzymes showed no dynamic change. On Day 3, the patient’s dyspnoea exacerbated with coldness in four extremities at 5 a.m. Vital signs showed HR 160 b.p.m., BP 99/67 mmHg, and SpO_2_ 96% under 8 L O_2_ mask. Lab work showed lactic acid 8.38 mmol/L (reference: 0.5–2.2 mmol/L). Arterial blood gas revealed pH 7.46, PCO_2_ 31.8 mmHg, PO_2_ 51.4 mmHg, and HCO_3_ 22 mmol/L. Chest X-ray revealed bilateral pulmonary oedema and pleural effusion. Intravenous amiodarone (900 mg) was given for AFRVR. He was transferred to the intensive care unit (ICU) under the impression of ADHF with cardiogenic shock caused by fluid overload, AFRVR (reduced LV filling), and severe functional MR with extremely impaired LV systolic function (insufficient forward stroke volume).

**Figure 1:**
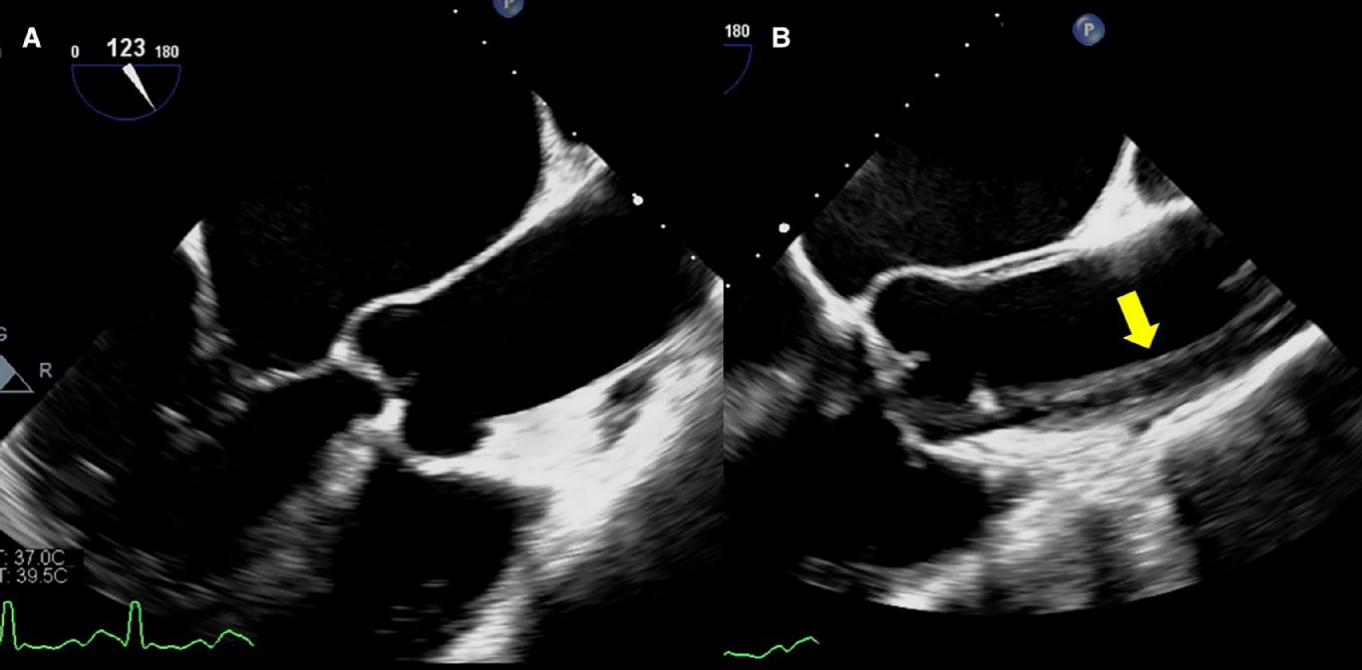
Transoesophageal echocardiogram (TEE) images. (*A*) The first mid-oesophageal long-axis view (123°) showed ascending aorta of 41 mm without evidence of dissection. (*B*) Twenty minutes after the probe insertion, the mid-oesophageal view (107°) revealed the presence of type A aortic dissection. The arrow indicated the dissected intimal layer.

**Figure 2:**
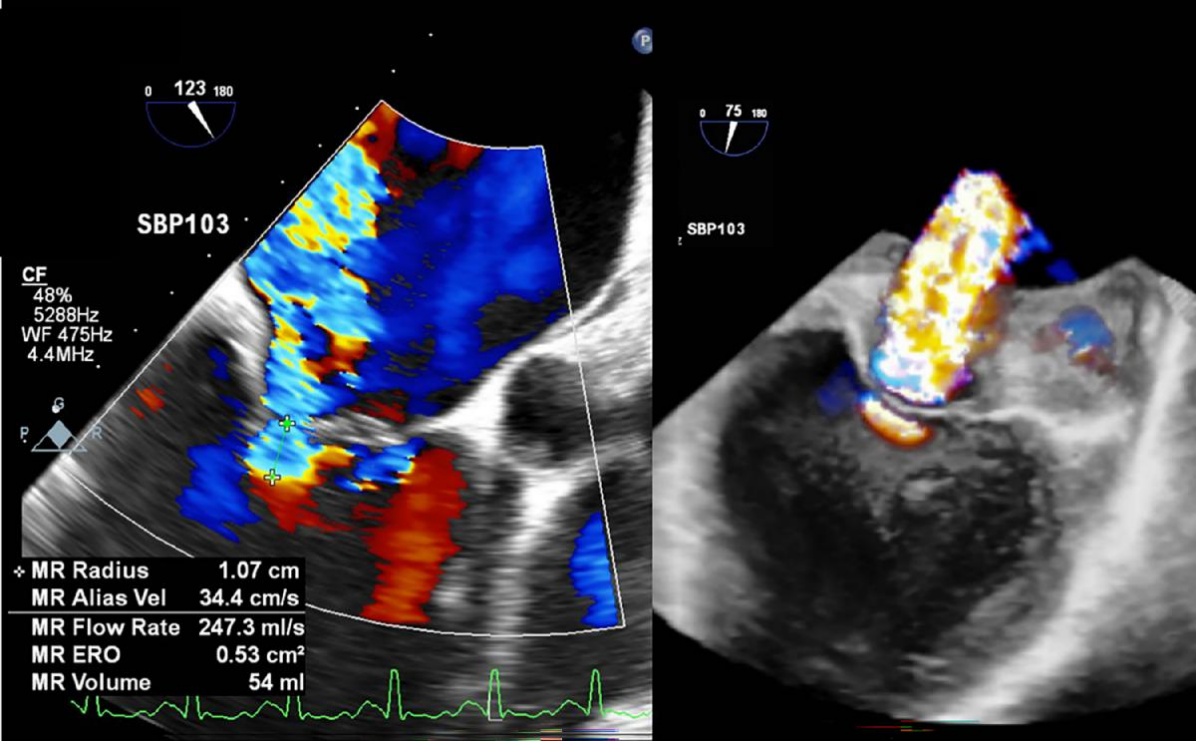
Transoesophageal echocardiogram (TEE) image for mitral regurgitation (MR). The TEE evaluation revealed MR (Carpentier type IIIB + I) with 2D effective regurgitant orifice (ERO) 0.52 cm^2^, 3D ERO 2.26 cm^2^, regurgitant volume 54 mL, and pulmonary vein reversal. The mitral valve (MV) structure was suitable for transcatheter edge-to-edge repair (MV area, 4.36 cm^2^; trans-MV mean pressure, 3.3 mmHg).

**Figure 3:**
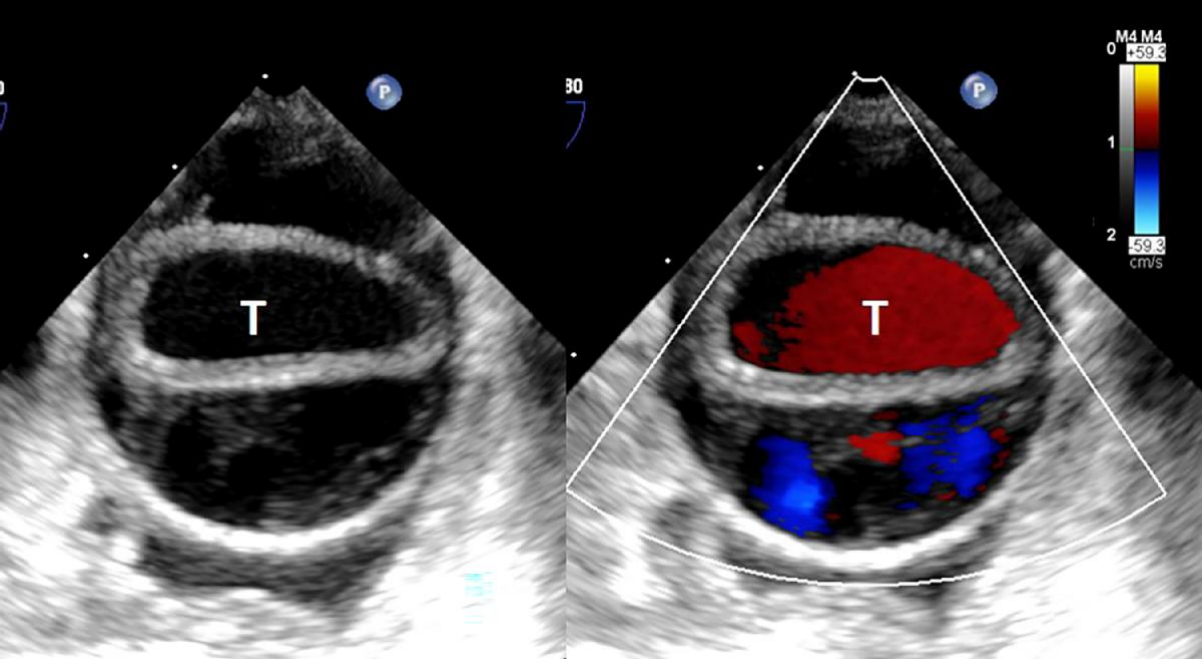
The type A aortic dissection. The aortic dissection extended to the descending aorta was revealed by transoesophageal echocardiogram with (right) and without (left) colour Doppler. The letter T indicates the true lumen of the affected vessel.

In ICU, the patient was intubated for hypoxic respiratory failure. Vital signs showed HR 152 b.p.m., arterial BP 92/75 mmHg (right radial artery), and SpO_2_ 98%. Twice synchronized direct current shock (50 J) converted AF to sinus rhythm (SR) with HR 82 b.p.m. and BP 92/69 mmHg. Dopamine infusion (4.3 μg/kg/min) was given. Given high surgical risk (STS score, 35.7%), M-TEER was considered the first choice. We were consulted for TEE to evaluate his eligibility. During TEE procedure, difficulties in probe insertion were encountered. We finally inserted the probe smoothly after giving a series of intravenous sedatives, including midazolam (15 mg), ketamine (50 mg), and cisatracurium (5 mg). Vital signs showed HR 111 b.p.m. (SR) and BP 99/67 mmHg under dopamine infusion (6.4 μg/kg/min). The first mid-oesophageal long-axis view (123°) showed a 41 mm mid-ascending aorta without evidence of dissection (*Figure 1A*) (see Supplementary material online, *Video S2*), as well as severe functional MR (effective regurgitant orifice area, 0.53 cm^2^, regurgitant volume, 54 mL with pulmonary vein reversal) (*Figure 2*). Twenty minutes after the probe insertion, TAAD appeared on the same view (107°) (see Supplementary material online, *Video S3*), extending from the aortic root, ascending aorta (46 mm; arrow in *Figure 1B*) to descending aorta (*Figure 3*). After recording the findings, we removed the probe immediately. Vital signs were HR 91 b.p.m., BP 97/54 mmHg under dopamine (4.3 μg/kg/min), and SpO_2_ 97%.

**Figure 4:**
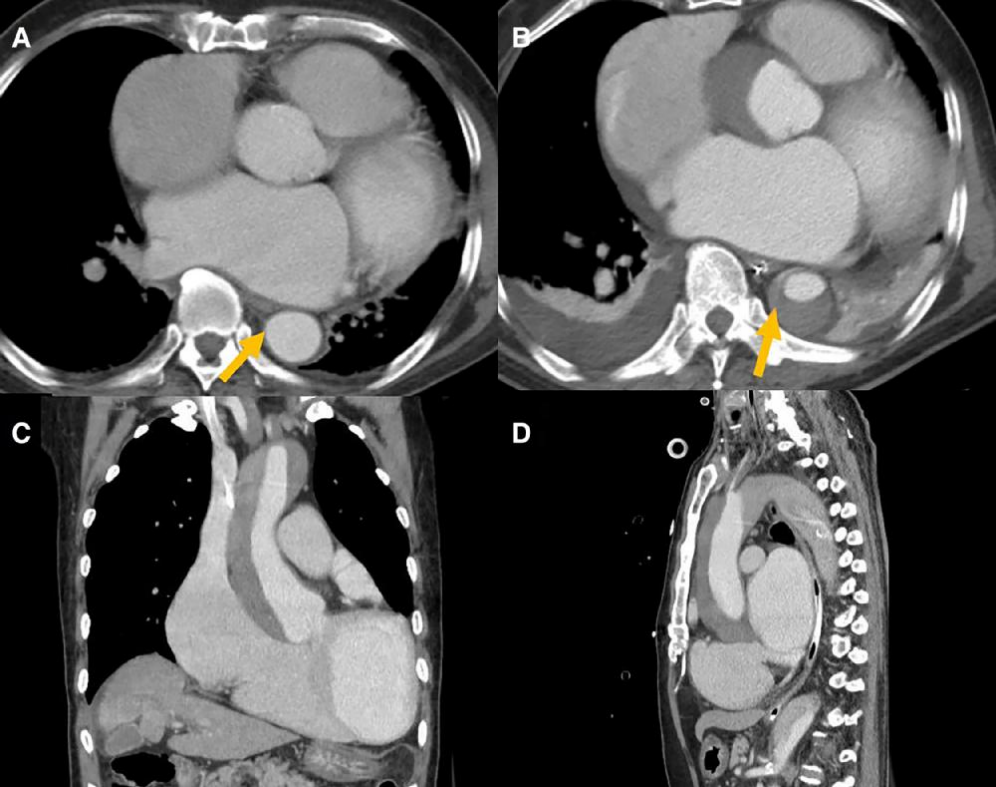
Computed tomography (CT) images before and after the transoesophageal echocardiogram (TEE) procedure. (*A*) The pre-procedural CT at the same level of suspected intimal flap, no signs of the dissection was noticed. (*B*) The axial view of the post-procedural CT showed the location of the suspected intimal flap, where contrast enhancement in the false lumen can be noticed (arrow). (*C*) Coronal view of the type A aortic dissection. (*D*) Sagittal view of the type A aortic dissection.

Contrast CT confirmed a TAAD from the aortic root to the infrarenal abdominal aorta (*Figure 4*). It also involved the innominate artery, bilateral common carotid arteries, and right renal artery. Our radiologist suspected that the intimal flap was located at the proximal descending aorta (*Figure 4B*, arrow). The CT 3 days before admission showed no intimal flap in the descending aorta (*Figure 4A*, arrow).

Due to the extremely high surgical risk of the TAAD repair, the patient received palliative care. On Day 4, his vital signs showed BP 98/47 mmHg under dopamine (8.6 μg/kg/min), RR 14 times/min, and SpO_2_ 96% under ventilator support. Yet, his consciousness deteriorated (Glasgow Coma Scale: E1M1VT); brain CT showed no intracranial haemorrhage. His condition went downhill rapidly despite the use of inotropes. Unfortunately, he passed away on Day 6.

## Discussion

From the literature review, we found only one case of newly diagnosed AD during TEE,^1^ a 79-year-old woman with paroxysmal AF. Under lidocaine and midazolam, she received TEE to assess left atrial appendage thrombi before AF catheter ablation. Five minutes after probe insertion, ascending aorta dissection occurred. She underwent aortic replacement surgery and survived uneventfully during nine months of follow-up.^1^

Several risk factors for AD have been recognized,^2^ including age > 65 years old, hypertension, smoking, aortic aneurysm, congenital disorder (e.g. Marfan syndrome and bicuspid aortic valve), or inflammatory disease (e.g. aortitis and giant cell arthritis). Our patient had advanced age and AAA but no history of hypertension or smoking.

Herein, we propose two mechanisms for AD. Firstly, a periprocedural Debakey type I dissection. Difficulties in probe insertion could impose stress to the patient, leading to a hypertensive surge.^1^ However, the evidence remained insufficient without continuous BP monitoring.

The second mechanism was a pre-procedural type B AD with retrograde TAAD, supported by our radiologist’s suspicion of an intimal flap at the proximal descending aorta (*Figure 4B*). In retrospect, the chest and back pain on second day might correlate with AD, especially in an old gentleman with AAA. Nevertheless, AD was not our prioritized differential diagnosis at that time; therefore, no corresponding examinations were arranged to verify the second possibility.

## Conclusions

According to the guidelines,^3^ contrast CT is the initial diagnostic tool for a suspected AD. However, our patient had poor renal function. Thus, TEE seems a reasonable alternative. Previous meta-analysis^4^ reported 98% sensitivity and 95% specificity for TEE in diagnosing thoracic AD. However, as might have occurred in our case, TEE may pose a risk of periprocedural AD. This creates a dilemma where experts should carefully assess the risks of kidney injury and AD. Four-limb BP may provide timely information for the diagnosis.^5^

In our case, the real mechanism of TAAD remained unknown. Nonetheless, the first TEE showed no signs of AD, implying that AD progressed or *de novo* occurred during the procedure. Hence, physicians must be vigilant about the possibility of TEE-related TAAD during the procedure, especially in patients at high risk of AD. Moreover, the risk for using TEE as an alternative diagnostic modality in AD with poor renal function should be carefully considered.

## Lead authors biography


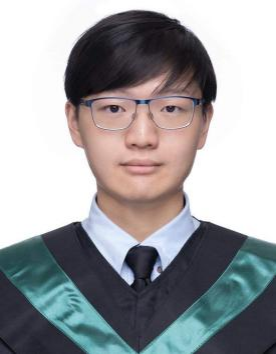


Chung-Yen Lee, a medical student at National Taiwan University, has a strong interest in cardiology and actively participates in clinical research during his NTUH clerkship. He aims to become an internal medicine physician in the future.


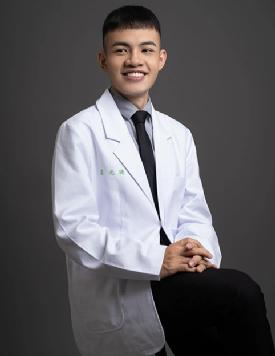


Kuang-Chien Chiang is a medical student at National Taiwan University. He has an enthusiastic mind for exploring new medical knowledge.

## Supplementary Material

ytae413_Supplementary_Data

## Supplementary material

**Consent:** The authors confirm that written consent for submission and publication of this case including images and associated text has been obtained from the patient’s next of kin in line with COPE guidance.

**Funding:** The study was supported by grants NSTC 113-2314-B-002-138 from the National Science and Technology Council of Taiwan and 113-IF0004, VN112-05 from National Taiwan University Hospital.

### Data availability

The data underlying this article are available in the article and in its online Supplementary material.
