# Supplementary material for: Type A aortic dissection during transoesophageal echocardiography: a case report
Source: Eur Heart J Case Rep. 2024 Aug 8;8(8):ytae413. doi: 10.1093/ehjcr/ytae413 (PMC11362548; doi:10.1093/ehjcr/ytae413)
Supplement: ytae413_Supplementary_Data [file ytae413_supplementary_data.zip › Supplemental material _legends.docx]

**Video S1. Computed tomography (CT) images of abdominal aneurysm.** The coronal views of abdominal CT images showed abdominal and bilateral iliac arterial aneurysms with remarkable tortuosity.

**Video S2.** **Transesophageal echocardiogram (TEE) video before type A aortic dissection (TAAD).** Mid-esophageal long-axis view (123˚) showed an intact proximal ascending aorta without evidence of dissection.

**Video S3.** **TEE video after TAAD.** Mid-esophageal long-axis view (107˚) showed TAAD with dissected intimal layer 20 minutes after probe insertion.
